# Supplementary material for: Activation of mGluR5 and NMDA Receptor Pathways in the Rostral Ventrolateral Medulla as a Central Mechanism for Methamphetamine-Induced Pressor Effect in Rats
Source: Biomolecules. 2020 Jan 16;10(1):149. doi: 10.3390/biom10010149 (PMC7022376; doi:10.3390/biom10010149)
Supplement: Supplementary file 1 [file biomolecules-10-00149-s001.pdf]

biomolecules

Figure S1

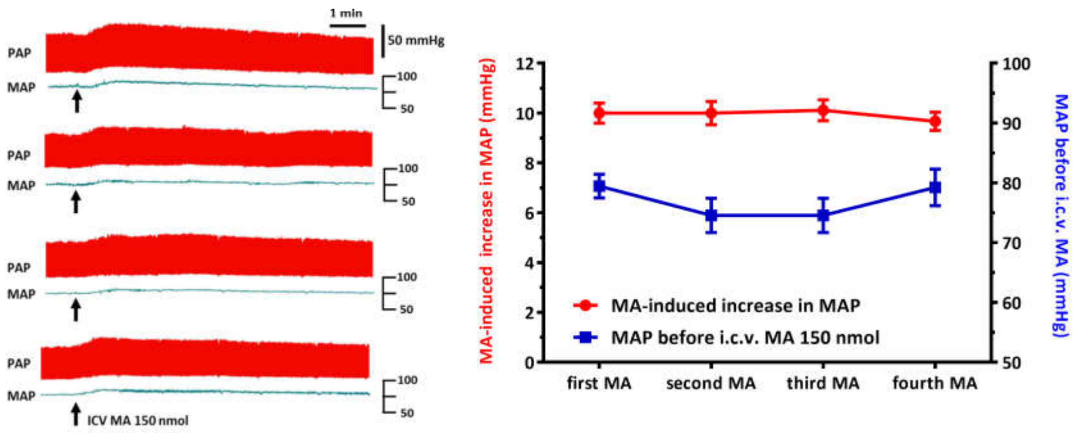

Figure S1. Left: representative recordings of pulsatile arterial pressure (PAP) and mean arterial pressure (MAP) showing the reproducible pressor effects induced by repeated application of MA. Arrows mark time of i.c.v. injections. MA (150 nmol) was applied at intervals of 30 min. Four sets of traces from top to bottom in sequence represent the pressor effects induced by MA in four consecutive injections in a SD rat. Right: line graph shows the amplitudes of MA-induced increase in MAP (red line and left y axis) and the MAP before every injection (blue line and right y axis). Values represent the mean ± S.E.M (n=9).
